# Naturally-occurring cholesterol analogues in lipid nanoparticles induce polymorphic shape and enhance intracellular delivery of mRNA

**DOI:** 10.1038/s41467-020-14527-2

**Published:** 2020-02-20

**Authors:** Siddharth Patel, N. Ashwanikumar, Ema Robinson, Yan Xia, Cosmin Mihai, Joseph P. Griffith, Shangguo Hou, Adam A. Esposito, Tatiana Ketova, Kevin Welsher, John L. Joyal, Örn Almarsson, Gaurav Sahay

**Affiliations:** 1 Department of Pharmaceutical Sciences, College of Pharmacy, Oregon State University, Robertson Life Sciences Building, 2730 Southwest Moody Avenue, Portland, OR 97201 USA; 2 Moderna Therapeutics, 200 Technology Square, Cambridge, MA 02139 USA; 3 French Family Science Center, Department of Chemistry, 124 Science Drive, Duke University, Durham, NC 27708 USA; 4 Department of Biomedical Engineering, Oregon Health and Science University, Robertson Life Science Building, 2730 Southwest Moody Avenue, Portland, OR 97201 USA

**Keywords:** DNA and RNA, Targeted gene repair, Oligo delivery

## Abstract

Endosomal sequestration of lipid-based nanoparticles (LNPs) remains a formidable barrier to delivery. Herein, structure-activity analysis of cholesterol analogues reveals that incorporation of C-24 alkyl phytosterols into LNPs (eLNPs) enhances gene transfection and the length of alkyl tail, flexibility of sterol ring and polarity due to -OH group is required to maintain high transfection. Cryo-TEM displays a polyhedral shape for eLNPs compared to spherical LNPs, while x-ray scattering shows little disparity in internal structure. eLNPs exhibit higher cellular uptake and retention, potentially leading to a steady release from the endosomes over time. 3D single-particle tracking shows enhanced intracellular diffusivity of eLNPs relative to LNPs, suggesting eLNP traffic to productive pathways for escape. Our findings show the importance of cholesterol in subcellular transport of LNPs carrying mRNA and emphasize the need for greater insights into surface composition and structural properties of nanoparticles, and their subcellular interactions which enable designs to improve endosomal escape.

## Abstract (web summary)

Endosomal sequestration of lipid-based nanoparticles is a barrier to delivery of nucleic acids. Here the authors test an array of cholesterol variants and perform in-depth investigation of nanoparticle shape, internal structure and intracellular trafficking.

## Introduction

The ascent of RNA drugs has bolstered the development of potential treatments for myriad undruggable disorders. Lipid nanoparticles (LNPs) are self-assembled nanostructures with the ability to encapsulate, protect, and deliver nucleic acids. LNP-mediated small-interfering RNA (siRNA) delivery has been clinically approved for the treatment of patients suffering from transthyretin-mediated amyloidosis (ONPATTRO^TM^)^1^. In recent years, RNA therapeutics have added another exciting new modality to its tool-box in the form of messenger RNA (mRNA). Pre-clinical and clinical studies are utilizing LNPs to package and deliver mRNA to induce rapid production of proteins for the treatment of infectious diseases, cancers, and rare genetic disorders, as well as for cellular and genetic manipulation^2–4^.

**Fig. 1:**
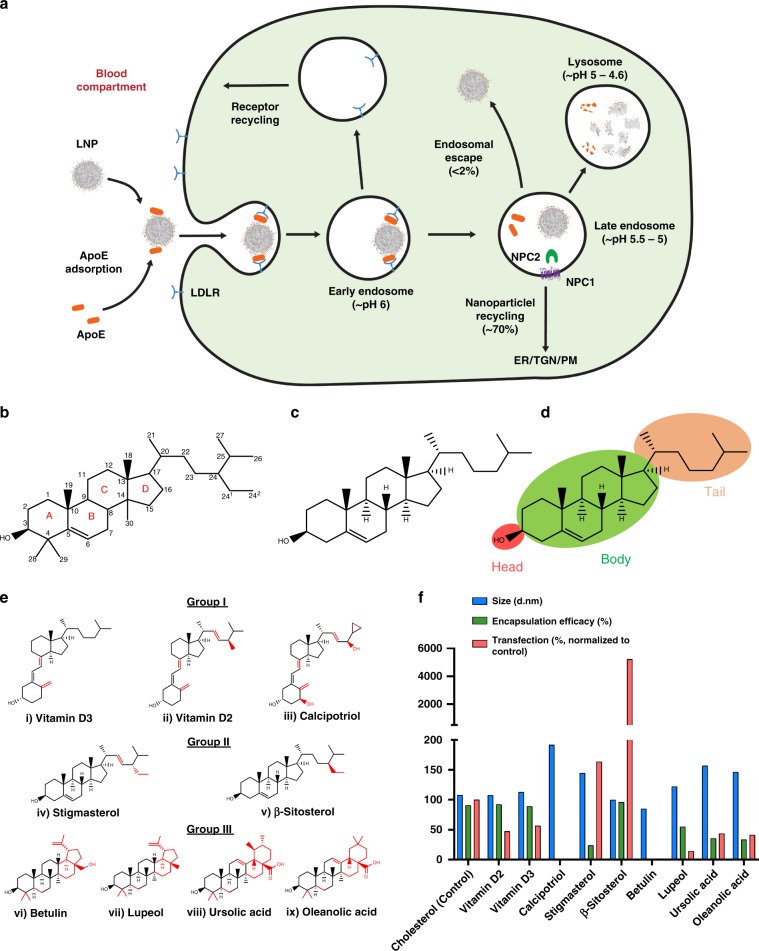
Cholesterol analogs screened for enhanced mRNA-based gene transfection. **a** Schematic showing desorption of PEG-lipids from LNPs allows ApoE binding causing LDL-mediated cellular uptake in cells following which a small portion of nucleic acids escape the endosome and the majority are recycled back by lysosomal transporters or directed to degradative endocytic compartments. **b** IUPAC-IUB nomenclature of (1989) cholesterol ring system with rings (A, B, C, and D), carbon numbering from C-1 to C-29, **c** the structure of cholesterol, and **d** schematic diagram showing classification of cholesterol into three regions: head, body, and tail. **e** Classification of cholesterol analogs- Group I-9,10-secosteroids, Group II-C-24 alkyl steroids, Group III-pentacyclic steroids. Structural variations from cholesterol are highlighted in red. **f** Cholesterol analogs were screened for particle size (nm), mRNA encapsulation (percent), and transfection efficiency (200 ng mRNA). Transfection experiments were conducted with *n* = 3. Source data are provided as a Source Data file.

The formulation of LNPs involves the rapid microfluidic mixing of an organic phase (composed of ionizable lipid dilinoleylmethyl-4-dimethylaminobutyrate (DLin-MC3-DMA), helper lipids (1,2-distearoyl-sn-glycero-3-phosphocholine (DSPC) and cholesterol), and 1,2-dimyristoyl-sn-glycerol-methoxy poly(ethylene glycol) 2000 (DMG-PEG2k)) with an acidified aqueous phase containing the nucleic acid. These nanoparticles organize into a core-shell structure, wherein the core contains the nucleic acid electrostatically complexed with the ionizable lipid, with cholesterol providing structural integrity. Meanwhile, DSPC and the PEG-lipid reside primarily on the surface along with a portion of the ionizable lipid and cholesterol forming the shell of the LNPs^5–7^. Research into the mechanisms underlying LNP delivery has revealed that following administration into the bloodstream, PEG-lipids on LNP surface are exchanged for serum proteins, including ApoE, which facilitates receptor-mediated cellular entry (Fig. 1a)^8^. It is suggested that protonation of the ionizable lipid within the LNPs at low endosomal pH promotes electrostatic interactions with the anionic endosomal membrane, which triggers the release of nucleic acid to the cytosol^9^. However, recent studies have demonstrated that most LNPs are trapped inside degradative endocytic compartments or are effluxed out of the cell, with only a small fraction (<2%) reaching the cytosol^10^. The process of endosomal escape remains one of the least understood and most uncooperative barriers to successful gene delivery^11^.

Much success towards enhanced nucleic acid delivery has been gained through screening or designing new ionizable lipids that can perturb the endosome^12–14^. Even helper lipids have not escaped investigation for maximizing endosomal egress^13,15,16^. For instance, the inclusion of cholesterol in nanoparticle formulations has been shown to improve efficacy, potentially due to enhanced membrane fusion^17,18^. It is hypothesized that cholesterol may exist in a crystalline form on the LNP surface, where it can promote endosomal escape^5,17^. Underscoring the magnitude of cholesterol’s influence on intracellular delivery, cholesterol, and cholesterol transport proteins have also been implicated to play a part in the cytosolic delivery of viruses and endocytic recycling/retention of nanoparticles^19–21^.

Drawing from this knowledge, herein, we decode the structural characteristics of cholesterol that are crucial for efficient intracellular delivery and improved gene transfection. A tremendous diversity of structural analogs of cholesterol exist as natural products (e.g., phytosterols that are plant-based sterols, which provide stability to the plant cell wall). We generate a collection of LNPs substituted with various natural cholesterol analogs to reveal the role of cholesterol structure on mRNA-based gene transfection. We identify a class of cholesterol analogs with an alkyl substitution at C-24, which, when incorporated inside an enhanced LNP (eLNP), can result in substantial increase in gene delivery. Moreover, these eLNPs are morphologically distinct than the traditional LNPs. The intracellular behavior of eLNPs is also different, suggesting that the structural aberrations and potentially altered surface lipid composition can lead to differential trafficking. A higher number of linear trajectories, as well as an increase in intracellular uptake and retention, are visualized. Through the use of cholesterol analogs, we can refine the nanoparticle structure, possibly arising from surface composition, leading to productive intracellular trafficking of nanoparticles, thereby enhancing cytosolic delivery.

## Results

### Screening LNPs formulated with cholesterol analogs

Cholesterol can be divided into three domains: head, body, and tail (Fig. 1b–d). The head of cholesterol is positioned in Ring A as an –OH group at C-3 tilted above the ring. In liposomal formulations, this –OH group is hypothesized to interact with the aqueous phase through its polarity and hydrogen bonding. The body consists of a fused four-ring system (1,2-cyclopentanoperhydrophenanthrene) containing three cyclohexane or cyclohexene rings (A, B, C), and one cyclopentane ring (D). The tail is made up of saturated alkyl side chain (C-20 to C-27) at position 17 of ring D (Fig. 1b–d)^22,23^. The right-handed conformation, saturation, and lack of functional groups in the tail are essential in allowing flexibility. The body and tail together contribute to the orderly formation of the hydrophobic lipid bilayer^23^. As such, cholesterol has been shown to increase the fluidity of gelled membranes, while inducing order to fluid membranes^24,25^.

For our initial screening, we selected three groups of analogs based on structural resemblances to the cholesterol ring system (For selection criterion, see Methods). Group I was comprised of Vitamin-D derivatives that differ from cholesterol in body with or without tail modification, Group II contained alkyl-substituted steroids that differ from cholesterol in the tail alone, and Group III featured cholesterol analogs wherein the tail was modified into a 5th ring (Fig. 1e). In later studies, we synthetically modified the head through conjugation of polar or non-polar groups.

The screening process was designed to evaluate two parameters: whether substitution of the aforementioned analogs supported packaging of mRNA inside a nanoparticle and whether they improved transfection efficiency (Fig. 1f and Supplementary Fig. 1). LNPs with cholesterol served as a positive control and had high encapsulation efficiency (94%) with a hydrodynamic diameter of ~100 nm. We found that Group I analogs failed to transfect efficiently following high (Vitamin D2, D3) or no (Calcipotriol) encapsulation. Despite observing a high encapsulation of mRNA for Vitamin D2 and D3-containing particles, the poor transfection suggests that the retention of the intact body region is essential for transfection. Among Group II analogs, stigmasterol showed minor improvement in transfection (1.6-fold relative to LNP) despite low-mRNA encapsulation. More importantly, β-sitosterol-substituted LNPs maintained comparable size and encapsulation but were considerably more effective at mRNA transfection (Fig. 1f and Supplementary Fig. 1). The potent cholesterol substitutes in Group II have a structural and stereochemical similarity at the C-24 ethyl group, which seems to have a substantial impact on boosting transfection efficiency. Previous studies have shown that in the multi-component model lipid membrane organization, C-24 alkyl group imparts minor defects in the ordering of lipid bilayer^26,27^. We hypothesize that these defects caused by C-24 ethyl group of Group II analogs might be responsible for the imperfections in the organization of lipids within LNPs. These imperfections may, in turn, facilitate intracellular uptake and cause efficient dissociation of LNPs during endosomal escape via membrane destabilization. The presence of double bond in stigmasterol between the C-22 and C-23 position restricts free rotation of the tail, potentially resulting in diminished mRNA encapsulation^27^. This disparity in the encapsulation and subsequent efficacy of stigmasterol may arise from the diminished lipid ordering capability of stigmasterol compared to β-sitosterol by virtue of the C-22 double bond^28^. Group III analogs showed limited encapsulation and poor transfection (Fig. 1e, f and Supplementary Fig. 1). The results of Group III showed that the presence of the 5th ring limits LNP activity. The possible steric hindrance arising from the presence of an additional 5th ring (either -cyclopentyl or -cyclohexyl) may interfere in the regular organization of lipid component and mRNA, leading to poor encapsulation and transfection ability of Group III analogs^29,30^.

### β-sitosterol enhances transfection

Furthermore, eLNPs were found to be more effective than LNPs after transfection of eight additional cell types (five human patient-derived lysosomal storage disease cell lines; J774A.1 and RAW264.7 mouse macrophages, as well as ex vivo apparently healthy human patient peripheral blood macrophages) (Supplementary Fig. 2). Furthermore, we formulated LNPs using 1,2-dioleyloxy-N, N-dimethyl–3-aminopropane (DODMA) as the ionizable lipid. DODMA-based eLNPs had up to a 48-fold improvement in gene delivery compared to control (Supplementary Fig. 3a). We further tested a biodegradable ionizable lipid (Heptadecan-9-yl 8-((2-hydroxyethyl)(4-(nonyloxy)−4-oxobutyl)amino)octanoate (Lipid 9)) that has been shown to have a higher transfection efficiency than MC3^14^. Similar to DODMA, lipid 9-eLNPs exhibited up to 32-fold and 6-fold improvement in expression over lipid 9-LNPs at 6- and 24-h time points, respectively (Supplementary Fig. 3b). The MC3-eLNPs were also able to enhance the delivery of Cas9 mRNA and siRNA that led to a 2.5-fold higher gene editing (frameshift introduced by non-homologous end joining) and up to a 6.5-fold improvement in gene silencing, respectively (Supplementary Fig. 3c, d). These findings suggest that C-24 alkyl-substituted cholesterol analogs can boost transfection independent of cell type, ionizable lipid, or nucleic acid cargo.

### C-24 alkyl derivatives enhance gene transfection

**Fig. 2:**
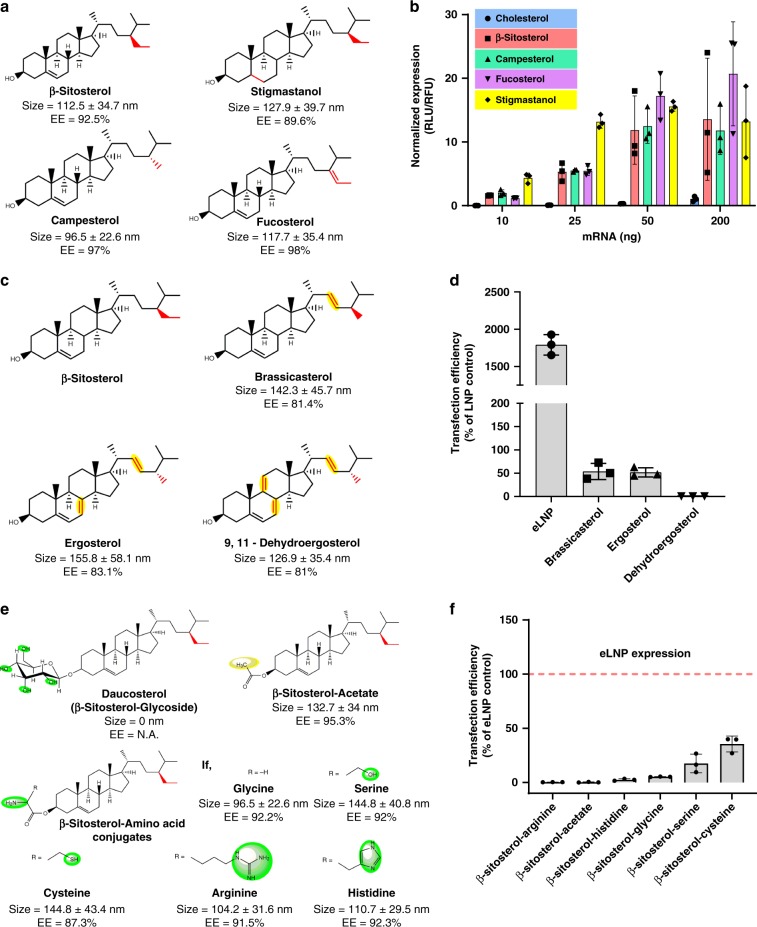
Structural features of C-24 alkyl derivatives that enhance gene transfection. **a**–**d** C-24 alkyl derivatives with variations in the tail and body were screened for their effect on size, mRNA encapsulation efficiency (*EE*), and transfection efficiency of LNPs (dosed at 10–200 ng mRNA per well for C-24 alkyl derivatives in **b** and 50 ng mRNA per well for sequential addition of double bonds in **d**). The variations of these structures from cholesterol and β-sitosterol are depicted as red and yellow, respectively. **e**–**f** Structural modification of the head group in β-sitosterol with either amino acids (polar) or acetate (non-polar) was also evaluated for its effect on nanoparticle size, encapsulation, and transfection of LNPs (200 ng mRNA per well). The color code is indicative of polarity (green), non-polarity (yellow), and structural difference from cholesterol (red). Horizontal dashed red line represents eLNP expression set to 100 percent. All experiments were conducted with *n* = 3; mean ± SD. Source data are provided as a Source Data file.

We next investigated whether other C-24 alkyl and alkenyl substitutes can improve LNP function. C-24 alkyl sterols belong to a versatile group of phytosterols that have a critical role in constituting the membrane composition and dynamics of plant cells. Previous studies have shown that the crystal defects induced by C-24 alkyl phytosterols are directly proportional to the length of the alkyl side chain (cholesterol <campesterol <β-sitosterol)^31^. Thus, we selected three additional phytosterols, namely stigmastanol (β-sitosterol analog with highly flexible body due to reduction of Δ−5 double bond in the sterol ring), fucosterol (restricted flexibility of ethyl group in the tail due to unsaturation) and campesterol (decreased length of alkyl substitution in the tail due C-24 methyl group) (Fig. 2a). These nanoparticles were comparable in size (~100 nm) to eLNPs and exhibited high encapsulation (>90%) (Fig. 2a) and outperformed cholesterol-based LNPs with an 11- to 211-fold improvement in transfection (Fig. 2b). These findings confirm the superiority of C-24 alkyl cholesterol analogs irrespective of the Δ−5 double bond and unsaturation or length of the C-24 alkyl chain (Fig. 2a, b).

Further, we were interested in studying the influence of body and tail rigidity of C-24 alkyl substitution on LNP function. We selected three more phytosterols, namely brassicasterol (low rigidity C-24 methyl analog with one double bond in tail region), ergosterol (moderately rigid C-24 methyl analog with one double bond in tail region and one double bond in body region) and 9, 11- dehydroergosterol (highly rigid C-24 methyl analog with one double bond in tail region and two double bonds in body region) (Fig. 2c). These nanoparticles were comparable in size (~100 nm) to eLNP and exhibited moderately high encapsulation (>80%) (Fig. 2c). To our surprise, all the analogs showed moderate (~50% for brassicasterol and ergosterol) or no transfection (dehydroergosterol) indicating the importance of flexibility in the body and tail region of C-24 alkyl cholesterol analogs (Fig. 2c, d). We can speculate that brassicasterol failed to encapsulate and transfect with the efficiency of other analogs in Fig. 2a, b, potentially due to the improper organization of different lipids in the LNPs^32^. Further, the sequential introduction of double bonds in the body region (Fig. 2c) may lower the membrane ordering capability of the sterols resulting in a drastic reduction of transfection efficiency (i.e., from ergosterol to 9,11-dehydroergosterol)^33^.

While we had studied the activity of the body and tail regions of sterols, we had yet to decipher the function of the head (C-3 hydroxyl) group. To define the role of the head group of β-sitosterol in LNP efficacy, we expanded our screening to include β-sitosterol analogs (Fig. 2e). Since only one natural C-3 analog of β-sitosterol (i.e., daucosterol, a β-sitosterol glycoside) was readily available, we synthesized a variety of synthetic C-3 analogs with polar (glycyl, arginyl, histidyl, seryl, and cystyl) and non-polar (acetyl) group conjugations (Supplementary Fig. 4) and tested their efficacy as LNP constituents. Interestingly, the polar substituent’s exhibited low transfection efficiency irrespective of encapsulation or size while arginine and the non-polar substituent (acetate) failed to transfect despite comparable size and encapsulation (Fig. 2e, f). We speculate that variation in polarizability at the C-3 position might result in reduced encapsulation and transfection. In addition to this, the –OH group is recognized by lysosomal transporters, which traffic cholesterol to the endoplasmic reticulum^34^. One can posit that modification of the –OH group leads to a differential rate of substrate recognition by cholesterol transporters causing reduction of transfection efficiency.

### β-sitosterol affects nanoparticle structure and morphology

**Fig. 3:**
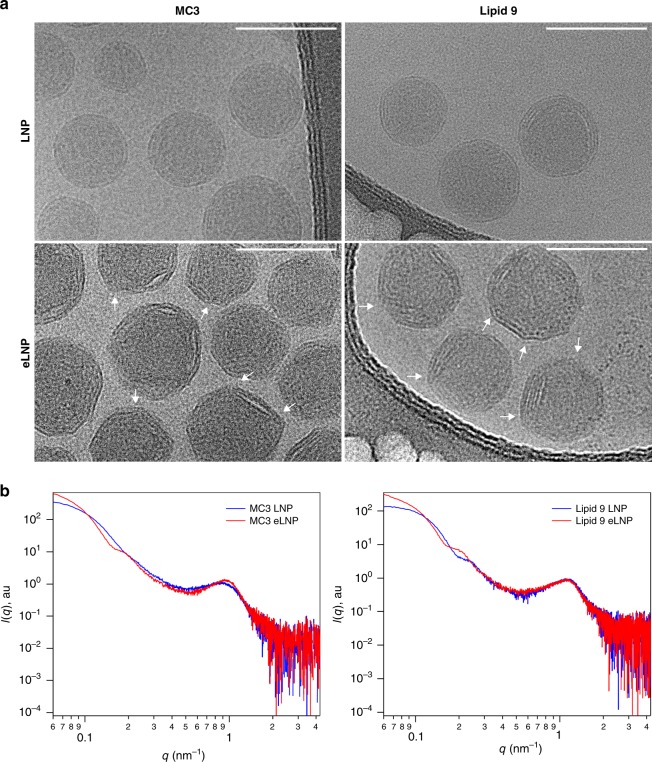
Impact of β-sitosterol on nanoparticle structure and morphology. **a** Cryo-TEM showing structural difference between LNP and eLNP (white arrows showing edges not observed in the spherical LNPs). Scale = 100 nm. **b** Small-angle X-ray scattering profiles of LNPs and eLNPs containing MC3 or Lipid 9.

The replacement of cholesterol with C-24 alkyl derivatives might result in the morphological or structural differences at the nanoscale, which may cause alterations in surface lipid composition, mRNA packaging, cellular uptake, intracellular trafficking, and its release, translating into enhanced gene delivery. We utilized cryo transmission electron microscopy (Cryo-TEM) to observe variation in structure and morphology between LNPs and eLNPs^5,6,35^. Both LNPs and eLNPs exhibit a core-shell structure from Cryo-TEM, showing a lamellar structure on the particle surface and a relatively amorphous particle core (Fig. 3a). Interestingly, unlike LNPs, where the surface exhibits a uniform curvature, the surface of eLNPs is highly faceted, which has been observed previously in liposomes^36–38^ and likely stems from phase separations of different lipid domains, e.g., ordered gel phase (*L*_β_) segregating from liquid disordered phase (*L*_α_). At the phase boundaries, a higher degree of disorder and packing defects is prevalent owing to the mismatch in the spontaneous curvature between different domains. It has been proposed that these defects could facilitate the fusion of nanoparticles with membranes^39–41^, thus resulting in better transfection efficiency.

To further investigate the internal structure of LNPs, we carried out small-angle X-ray scattering (SAXS) experiments, which have been commonly employed to study the organization of nucleic acids encapsulated in polymeric or lipidic systems^42–45^. Figure 3b and Supplementary Table 1, respectively, shows the SAXS profiles and data collection parameters of MC3 and lipid 9 nanoparticles. Guinier approximation using the low *q* data reveals that the radius of gyration (*R*_g_) is larger for MC3 eLNPs than LNPs (Supplementary Fig. 5 and Supplementary Table 2), corroborating the dynamic light scattering (DLS) results (Supplementary Table 3). Moving to the medium *q* range, the broad peak at *q* = 0.2 nm^−1^ is the form factor of the core-shell structure of MC3 eLNPs, the lack of which for MC3 LNPs suggests that the nanoparticle core is likely more polydispersed. Consistent with the Cryo-TEM data, both MC3 eLNPs and LNPs show a peak at *q* ~ 1 nm^−1^ characteristic of an organized lamellar phase with mRNAs either associated with one bilayer, forming a single lamellar structure, or sandwiched between bilayers, presenting a multilamellar phase. Moreover, MC3 eLNPs have a more regular lamellar phase as indicated by the sharper peak than MC3 LNPs. The lamellar spacing, calculated as $d = \frac{{2\pi }}{q}$, is estimated to be 6.64 nm for MC3 eLNPs, which is slightly smaller than the *d*-spacing of 7.22 nm obtained for MC3 LNPs. The lamellar *d*-spacing is a sum of the thickness of a lipid bilayer and the width of mRNA molecules. A smaller *d*-spacing in MC3 eLNPs may result from a slightly tighter packing of lipids and mRNAs. We further compared the SAXS profiles of LNPs and eLNPs composed of the biodegradable lipid, lipid 9, and found that the difference in internal structure (e.g., core and shell) for lipid 9 was not as evident as that of MC3 containing nanoparticles except for the *R*_g,_ which is larger for lipid 9 eLNP (Fig. 3b, Supplementary Fig. 5, and Supplementary Table 2). This implies that the internal structure of the nanoparticles probed by SAXS, such as mRNA organization, may not be critical for the improved biological activity of eLNPs. On the other hand, the unique surface morphology of eLNPs observed using Cryo-TEM presents a possible explanation for their enhanced transfection. More in-depth surface characterizations are necessary in the future to build a better understanding of the relationship between biological activity and physical property.

### β-sitosterol modulates intracellular trafficking

**Fig. 4:**
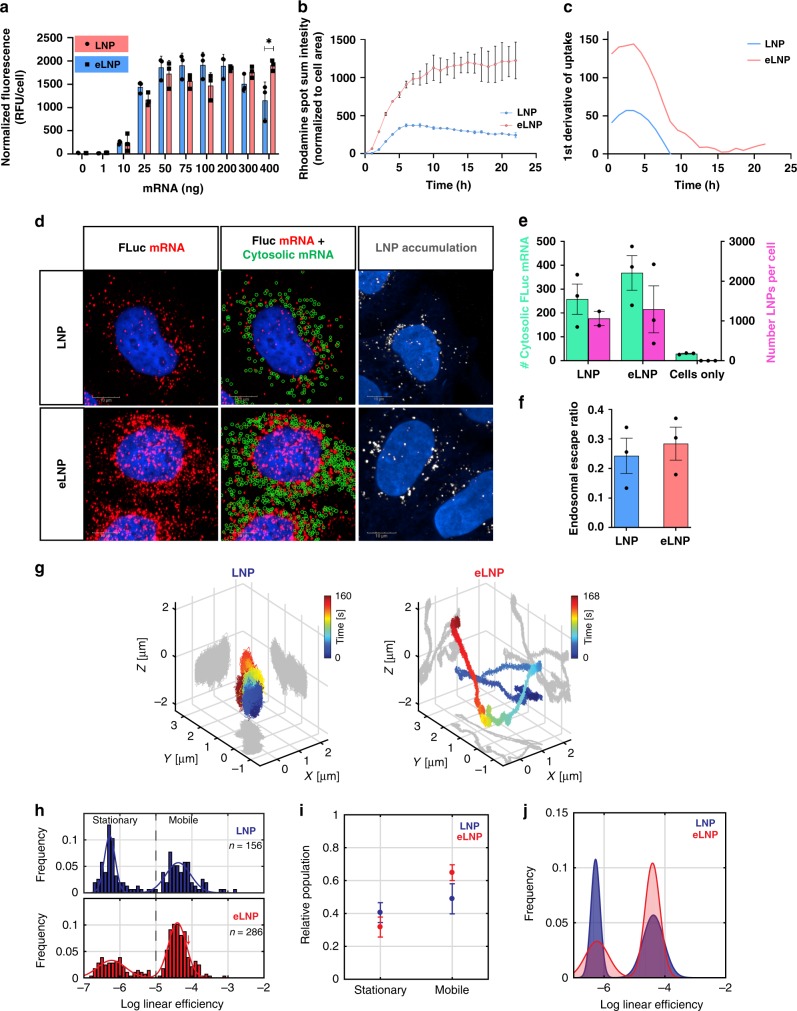
Intracellular delivery and endosomal escape of nanoparticles. **a** HeLa cells were exposed to β-sitosterol containing eLNPs at different doses (1–400 ng) and compared to cholesterol-containing LNPs for cellular uptake. Cy5-EGFP mRNA was used as a tracer, and high-content imaging was performed and quantified using the Cellomics software. (*n* = 3; mean ± SD. *p ≤ 0.05, **p ≤ 0.01, ***p ≤ 0.001. Significance was determined using nonlinear regression with ANOVA.) Source data are provided as a Source Data file. **b** Uptake kinetics of LNPs and eLNPs at 10 ng mRNA per well (0–24 h) in HeLa cells. **c** 1st derivative of uptake of LNP and eLNP demonstrates substantially higher rate of uptake and retention in HeLa over the 24 h post-transfection. **d** Endosomal escape was visualized using smFISH. Representative fluorescent images showing mRNA, LNP, and image analysis after delivery with LNP or eLNP in HeLa cells. **e** Quantitative image analysis of the number of cytosolic mRNAs (turquoise bars) compared to the number of LNPs per cell (magenta bars) after cellular delivery. **f** Ratio of cytosolic mRNA delivery to total LNP uptake indicative of endosomal escape. **g**–**j** 3D-DyPLoT was performed for high spatiotemporal resolution of nanoparticle transport. **g** Representative trajectories of internalized LNP and eLNP. The measured 3D position is plotted at 1 ms temporal resolution. **h** Histograms of the log-linear efficiency for LNP (blue) and eLNP (red), both of which display a bimodal distribution of stationary and mobile. **i** Population of the stationary and mobile fractions for LNP and eLNP. Population was measured by fitting the histograms in **h** to a two-term Gaussian and extracting the area under the two modes of the distribution. *n* = 156 (LNP) and 286 (eLNP); mean ± SD. *p* = 0.0009. Significance was determined using *t*-test. **j** Best fit distributions of LNP and eLNP, showing a clear increase in the mobile fraction for eLNP.

To interrogate whether these structural differences affect the intracellular transport of these nanoparticles, we deployed state-of-the-art imaging techniques. High-content imaging of LNPs and eLNPs containing a labeled mRNA as a tracer showed no difference in overall uptake, except at 400 ng where eLNPs showed significantly higher cellular uptake at 24 h post-transfection (Fig. 4a). Using a high-resolution automated confocal microscope, we further measured the kinetics of uptake (nanoparticles containing rhodamine-DOPE) and visualized the cytosolic localization of mRNA utilizing stellaris single-molecule fluorescence in situ hybridization (smFISH). We found that eLNPs show a higher rate of uptake and retention within cells as compared to LNPs over 24 h (Fig. 4b, c). Rapid uptake of LNPs and eLNPs was observed in the first 4 h, followed by a slow-down in the rate of internalization of eLNPs, whereas LNP retention appears to be decreasing (Fig. 4b, c). Previously, it has been shown that a cholesterol transporter residing on the surface of late endosome causes efflux of LNPs thus reducing their efficacy^21^. It is possible that the replacement of cholesterol with sitosterol leads to reduced recycling of eLNPs thus causing higher cellular retention and improved gene expression. Utilizing smFISH to visualize endosomal escape, we used fluorescent probes with a high degree of sequence complementarity capable of visualizing single strands of target mRNA, encapsulated inside a vesicle or free in the cytosol. We applied this technology in conjunction with a recently developed method that used electroporated mRNA as a standard for the fluorescent intensity of a single cytosolic mRNA molecule, whereas entrapped mRNA will produce a more intense signal because it will be clustered within an LNP and/or endosome (Fig. 4d and Supplementary Fig. 6a). At an early time-point (4 h), a higher average, albeit non-significant, uptake of nanoparticles and endosomal escape of mRNA was observed (Fig. 4e). The subsequent efficiency of endosomal escape (ratio of cytosolic mRNA to total intracellular LNP) of eLNP-delivered mRNA was on average higher but not significant compared to LNPs at the 4-h time-point (Fig. 4f). Enhanced retention of eLNPs in vesicular compartments likely leads to a higher total cytosolic mRNA over time. Since endosomal escape is a transient event, we deployed three-dimensional (3D)-Dynamic Photon Localization Tracking (3D-DyPLoT) that is capable of measuring intracellular transport of individual nanoparticles in live cells on a millisecond temporal resolution^46,47^. 3D-DyPLoT is a real-time 3D single-particle tracking method that locks onto moving fluorescence targets and tracks their 3D position with high spatiotemporal precision. To track the differential trafficking of LNP and eLNP loaded with Cy5-labeled mRNA, particles were incubated with HeLa cells for 1 h, and the motion of internalized particles was captured for up to 3 h from the start of the incubation. Representative trajectories for LNP and eLNP are shown in Fig. 4g and Supporting Movie in 3D space. As endosomal trafficking is directed along the microtubule network^48^, each trajectory was analyzed for linear transport based on a sliding window linear efficiency^49^. The linear efficiency was calculated for *n* = 156 LNPs and *n* = 286 eLNPs (Fig. 4h). Two things stand out from this analysis. First, both the LNP and eLNP particles display a bimodal transport efficiency, with characteristic stationary and mobile phases at approximately the same linear efficiency for both particles (Fig. 4h). Second, the mobile phase of eLNP is significantly more populated than for LNP (Fig. 4i) with a clear shift in eLNP trajectories towards higher mobility in contrast to LNP (Fig. 4j), indicating an increase in directed cargo transport of internalized eLNPs relative to internalized LNPs. This phenomenon can be either due to higher endosomal escape that allows eLNPs to diffuse freely in the cytosol, or an indication that eLNPs might exploit non-degradative trafficking pathways resembling a pearl-on-a-string motion, which often leads to a productive site for endosomal escape^50^. These enhancements could be due to the structural or surface lipid composition differences in eLNPs, which facilitate intracellular delivery, endosomal escape or differential interactions of sitosterol with signaling components that drive eLNPs to more productive sites that increase endosomal escape.

### Effect of defective cholesterol trafficking

**Fig. 5:**
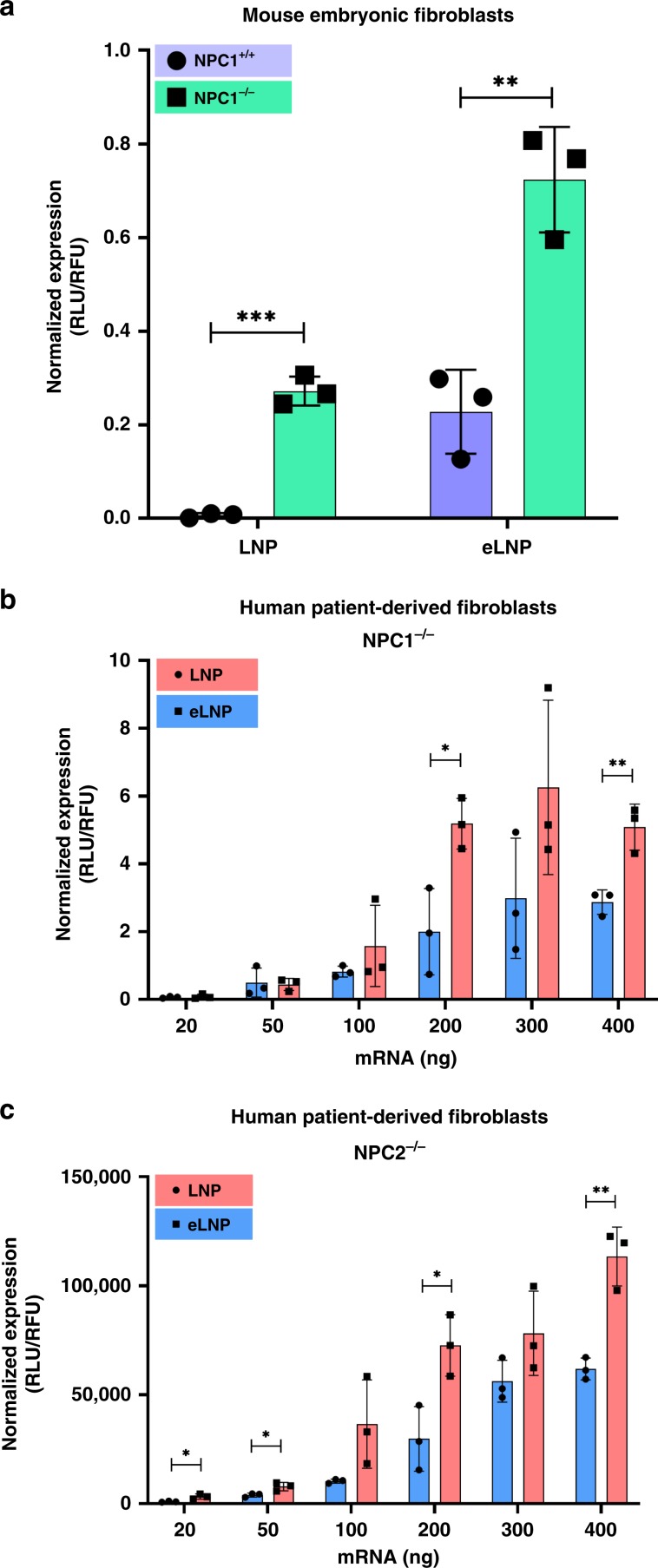
Effect of defective cholesterol trafficking on eLNP-mediated gene transfection. LNPs and eLNPs were compared for their ability to deliver mRNA to **a** wild-type (WT) and NPC1 knockout (NPC1^−/−^) mouse embryonic fibroblasts (200 ng mRNA per well), and **b** patient-derived NPC1, and **c** NPC2 loss-of-function fibroblasts. All experiments were conducted with *n* = 3; mean ± SD. (*p ≤ 0.05, **p ≤ 0.01, ***p ≤ 0.001. Significance was determined using multiple *t*-test.) Source data are provided as a Source Data file.

Exogenous cholesterol is delivered to the cell to maintain cellular homeostasis through low-density lipoprotein (LDL)^51^. LDL particles package esterified cholesterol inside a non-polar core and eventually localize to the LE/Ly, where cholesterol is de-esterified^51,52^. Two partner proteins, Niemann Pick Type C-1 (NPC1) and Niemann Pick Type C-2 (NPC2), disassemble LDL and mediate escape of cholesterol from LEs to the cytosol, endoplasmic reticulum or the plasma membrane^34,53^. NPC2 is a soluble protein that binds to the hydrophobic tail of cholesterol and presents the head (i.e., –OH) to the N-terminal domain (NTD) of NPC1, a transmembrane protein that resides on the surface of lysosomes shunting cholesterol out^54,55^. It has been shown that ~70% of internalized nanoparticles are recycled from the late endosomes/lysosomes to extracellular space in an NPC1 directed process^21^. The absence of NPC1 led to enhanced retention of nucleic acids in late endosomes/lysosomes, which translated into improved cytosolic delivery. Furthermore, the intracellular trafficking of C-24 alkyl derivatives has been shown to be restricted in the endosome^56^. These studies led us to hypothesize that the substitution of nanoparticles with cholesterol analogs may modulate NPC1/2 activity, thus reducing eLNP efflux and consequently improving intracellular availability and mRNA delivery. This observation is further reinforced by the higher retention of eLNPs compared to LNPs in HeLa cells (Fig. 4b, c). We utilized cell lines with loss-of-function mutations in NPC1 or NPC2 to investigate this possibility (Fig. 5). We found that cellular deficiency in NPC1 resulted in enhanced transfection for both, LNP and eLNP, with eLNP again demonstrating superior efficacy (Fig. 5a, b). NPC2-deficient cells responded with lower relative enhancement in eLNP-mediated gene delivery (Fig. 5c). These studies show that defects in endosomal recycling contribute to the enhanced endosomal escape of nanoparticles. The differential binding of C24 alkyl derivatives with the endosomal efflux machinery may lead to higher retention of eLNPs^56^. The exact role of surface morphology on interactions of eLNPs and LNPs with these partner proteins remains unclear.

## Discussion

A biologically essential molecule, cholesterol is primarily vital for maintaining cell membrane integrity. Cholesterol homeostasis is carefully regulated in the human body. Biosynthesis of cholesterol mainly occurs in the liver through a multi-step enzymatic process and can serve as a precursor to the formation of steroids, bile acid, and Vitamin-D^51^. Cholesterol is also available as part of dietary intake wherein it is converted into an esterified form in the intestinal tract, incorporated into low-density and very-low-density lipoprotein (LDL, VLDL) particles and directed to the liver via the bloodstream^57^. LDL and VLDL bind a serum protein, ApoE, that targets them to the LDL receptor, highly expressed on the hepatocytes. LDL is then engulfed in a clathrin-coated pit that directs it into the late endosome/lysosomes (Fig. 1a). Here, NPC2 strips LDL of cholesterol-ester, which is de-esterified by acid lipase before being handed over to NPC1, residing on the late endosome/lysosomes, for efflux (Fig. 1a)^51^. The efflux of cholesterol from the lysosomes remains controversial and an active area of research^21^. In recent years, it has been shown that cholesterol can activate mTOR, which resides on the lysosomal surface and is responsible for protein synthesis and ribosomal biogenesis^58^. As LNPs have a high-cholesterol content, it is intuitive that they follow a similar itinerary from being captured by ApoE and then processed via the LDL pathway (Fig. 1a)^8^. The downstream processes that are involved in the endosomal processing of cholesterol have also been shown to take part in LNP trafficking^21^. A case in point is that either through blocking or absence of NPC1, higher efficiency of nucleic acid delivery is achieved by preventing these particles from reaching non-productive sites. More recently, synthetic analogs of cholesterol have been used to show that its structure is important for effective in vivo delivery^59^.

The importance of cholesterol has not gone unnoticed, stimulating decades of research on lipid-based delivery systems^18,60,61^. By the addition of varying molar ratios of cholesterol, a higher efficacy in DNA delivery has been reported^18^. Recent studies posit that the lower solubility of cholesterol in the core region may lead to its enrichment on the LNP surface, promoting its crystallization and potentially contributing to endosomal fusion^5^. We were able to show that phytosterols, which are C-24 alkyl derivatives can enhance nucleic acid delivery (Fig. 2a, b). Phytosterols are part of our dietary intake, and the absorption and metabolism of these phytosterols have been investigated by several groups^62,63^. β-sitosterol, the primary analog uncovered through our study, has been reported to have potential health benefits such as protection from cardiovascular diseases and anti-cancer activity^64,65^. Remarkably, the mere addition of a methyl or ethyl group at the C-24 position renders a considerable impact on the morphology, as well as the intracellular uptake and retention of LNPs, resulting in a dramatic improvement in efficacy (Figs. 1e, f,  2a, b,  3a,  4b, c,  5 and Supplementary Figs. 2 and  3). The structural analysis of the LNPs and eLNPs by X-ray scattering and Cryo-EM experiments revealed interesting details, which suggest that eLNPs have a different surface morphology (Fig. 3 and Supplementary Fig. 5). The diminished membrane ordering capability of C-24 alkyl derivatives of cholesterol is well documented^28,31,66,67^. As such, the surface of nanoparticles containing β-sitosterol may have higher defects, which might contribute to improved fusogenic properties (Fig. 3a). The changes in the surface morphology of the nanoparticle may also reflect changes in the surface lipid content, which interacts with the endosomal lumen and facilitates enhanced fusion with the endosome. The surface composition of LNPs has been previously demonstrated to impact their delivery efficiency^5^. Furthermore, we were able to visualize the intracellular trajectories of the nanoparticles and found more linear trajectories associated with eLNPs, suggesting differential endosomal trafficking that results in the movement of a portion of these nanoparticles through non-degradative pathways (Fig. 4g–j). These routes perhaps are more productive routes that enable higher cytosolic delivery of mRNA. Our other hypothesis is based on a study from the mid ’90 s that showed liposomes bearing C-24 alkyl derivatives of cholesterol tend to accumulate in lysosomal compartments^56^. We find higher retention of eLNPs, giving a higher probability of eLNPs to interact with the endosomal compartment and cause escape. This model is supported by earlier findings that enhanced endosomal retention due to defective recycling boosts siRNA delivery^21^.

Also, we do find that the scale-up of nanoparticle formulations, variability in batch quality amongst phytosterols, purification process of the nanoparticle formulation, and cell types led to variability in the fold-differences when comparing LNPs with eLNPs. Future studies may require optimization of formulation parameters to reduce variability. Additionally, lipid-9, a more potent lipid designed for endosomal escape, demonstrated a substantially higher gene transfection in an eLNP as compared to MC3-eLNP (Supplementary Fig. 3b), suggesting that the use of cholesterol analogs leads to the modulation of nanoparticle packaging for enhanced intracellular delivery but the ionizable lipid might still be the main ingredient that drives transfection efficiency.

Overall, through our studies, we were able to show that enhancement in intracellular delivery may be related to nanoparticle surface composition and structure, and their subsequent subcellular interactions. More work is required in the future to better understand the importance of LNP surface lipid content, structure, and its role in biological activity. LNPs and eLNPs may follow a similar initial uptake pathway after which the ionizable lipid becomes protonated and binds electrostatically with the endosomal membrane causing the escape of nucleic acids. The superiority of eLNP may arise from its increased fragility once inside the cells due to the incorporation of β-sitosterol. This enhanced fragility, originating from an altered surface composition and shape, perhaps promotes eLNP fusion with the endosomal membrane. It is possible that eLNP shape and surface lipid composition leads to differential trafficking that drives it to privileged pathways more suitable for escape from the endosomes. It is probable that higher retention of eLNPs due to a decrease in its interactions with lysosomal transporters provides them enough residence time to escape from the endosomes subsequently causing enhanced nucleic acid delivery. Whether the structural characteristics, surface lipid content, and interactions with the cells can improve gene delivery in vivo remains to be explored. Understanding the nanoparticle design, controlling its structure and its surface lipid composition can enable non-viral vectors to breach this most formidable intracellular barrier—the endosome.

## Methods

### Chemicals

All Fmoc-protected amino acids were purchased from Peptide International Inc. β-sitosterol (95–97%), fucosterol, 2,3-Dioleyloxy-1-(dimethylamino) propane (DODMA), heparin sodium salt, N-hydroxy succinimide (NHS), N-(3-Dimethylaminopropyl)-N′-ethylcarbodiimide (EDC), piperidine, trifluoroacetic acid, acetonitrile, dimethylformamide (DMF), dichloromethane (DCM), and ethanol were purchased from Sigma-Aldrich. Acetic anhydride was obtained from Fischer Scientific Inc. All cholesterol analogs were purchased from Cayman Chemicals. β-sitosterol (90%) for conjugation was obtained from Abcam. Stigmastanol was purchased from Matreya LLC. Paraformaldehyde was purchased from Electron Microscopy Sciences. Triton™ X-100 was purchased from Acros Organics. Quant-iT™ RiboGreen® RNA reagent and rRNA standards were purchased from Life Technologies. CellTiter Fluor Cell Viability Assay and One-Glo™ Luciferase Assay was purchased from Promega. Cyanine-5 tagged EGFP mRNA, and Cas9 mRNA was purchased from TriLink Biotechnologies. Firefly luciferase (fLuc) mRNA was either gift from Moderna or purchased from Trilink Biotech. DLin-MC3-DMA, DODMA, cholesterol, DSPC, DMG-PEG2k were either obtained from Moderna Therapeutics, BioFine International or Avanti Polar Lipids. Lipid 9 was a gift from Moderna Therapeutics and its structure has been published elsewhere^14^. Stealth RNAi™ siRNA Luciferase Reporter Control was purchased from ThermoFisher Scientific. All cell culture reagents were purchased from Corning.

### Selection of cholesterol analogs for screening

The analogs were selected based on the structural resemblance with that of cholesterol ring (1,2-cyclopentanoperhydrophenanthrene ring) and classified into three major groups (I–III) as

I. 9, 10-Secosteroids-Variation in the ring structure in B, as well as in the side chain starting from C-20 to C-27. [Secosteroids - broken steroid]. These all are derivatives of Vitamin-D.
II. C-24 alkyl steroids-Variation in the side chain starting from C-22 to C-24 only. These are dietary phytosterols.
III. Pentacyclic steroids-cholesterol analogs containing five rings.

The structural variations of the selected analogs with respect to cholesterol are discussed below in detail. The structural explanations are according to the IUPAC-IUB nomenclature of the sterol ring system (Fig. 1b, i)

Group I: This group consists of Vitamin-D derivatives, which are structurally 9, 10-Secosteroids. The 9, 10- Secosteroids were formed by breakage of the bond between C-9 and C-10 and further resonance rearrangement to form structures with a deformed Ring B. The remaining rings like A, C, and D remain intact, similar to that of Cholesterol. Vitamin D3 has an identical structure with that of cholesterol from C-20 to C-27. Vitamin D2 has a double bond between C-22 and C-23, as well as a C-24 methyl group. We also selected calcipotriol with deformed ring B, a double bond between C-22 and C-23, two additional –OH groups (C-1 and C-24) and an additional bond between C-26 and C-27 to form a cyclopropane ring at the side chain. The selection of calcipotriol was assumed to reveal how additional polar groups and cyclopropane ring in the side chain will affect the efficacy of LNPs. The biocompatibility of these derivatives has also played a crucial role in the selection methodology.

Group II: This group consists of C-24 α-alkyl sterols (namely ethyl group) with intact rings A, B, C, and D with that of cholesterol. They are stigmasterol and β-sitosterol. In stigmasterol, there is an additional double bond between C-22 and C-23 in addition to a C-24 ethyl group. Both analogs are phytosterols that are widely distributed in the plant kingdom. These are major dietary sterols rich in fats or oils of soybean, nuts, vegetables, etc. The stereochemical orientation (α-alkyl sterol) has a phylogenetic significance. 24-β-alkyl sterols are found in lower organisms such as protozoa, fungi, whereas 24-α -alkyl sterol isomers predominate with other sterols in advanced organisms like vascular plants (e.g., Phytosterols like Stigmasterol and β-sitosterol) and have more dietary significance in human beings.

Group III: This group consists of cholesterol analogs, which consist of five rings. Here, the side chain of cholesterol from C-20 to C-27 was rearranged along with ring D to form the additional ring. This group includes Betulin and Lupeol where the ring is five-membered, as well as ursolic acid and oleanolic acid where the ring is six-membered. There exist polar groups like –COOH and –OH in addition to the hydroxyl group at C-3. In all the above analogs C-4 has dimethyl substitution with the reduction of C-5 and C-6 double bond. In ursolic acid and oleanolic acid, there exists a double bond between C-12 and C-13. All the analogs of group III mentioned above are natural pentacyclic triterpenoids while cholesterol is a tetracyclic triterpenoid. They were selected to investigate the role of the alkyl side chain (C-20 to C-27) of cholesterol in LNP formulation.

All the nine analogs selected for our initial screening were either naturally occurring phytosterols or vitamin-D derivatives, which are highly biocompatible and biodegradable.

### Formulation and particle characterization

LNPs containing ionizable lipid (DLin-MC3-DMA, Lipid 9, or DODMA):sterol:DSPC:DMG-PEG2k at molar ratios of 50:38.5:10:1.5 were formulated using NanoAssemblr Spark or Benchtop (Precision NanoSystems). A lipid mix concentration of 5.5 mM was used. Total flow rate was maintained at 9 mL per min at a 3:1 ratio of aqueous to organic phase for formulating on the Benchtop. NanoAssemblr Spark functions at a constant flow rate and 3:1 ratio of aqueous phase to organic phase. A N:P ratio of 5.67 between ionizable lipids and the nucleic acids was maintained throughout the study. The nanoparticles were buffer exchanged with phosphate-buffered saline (PBS; (pH 7.4) and concentrated using Amicon Ultra centrifugal filters and characterized for hydrodynamic diameter using DLS and encapsulation efficiency using modified Quant-iT RiboGreen assay. Nanoparticles were diluted in PBS and hydrodynamic size of the nanoparticles was measured using DLS on Malvern Zetasizer Nano. To determine mRNA encapsulation efficiency, nanoparticles (or PBS, blank) were diluted in TE buffer to achieve a concentration of 2–4 ng per µL mRNA per well. These samples were aliquoted and diluted 1:1 in TE buffer (measuring unencapsulated mRNA) or TE buffer with 2% Triton X-100 (measuring total mRNA). Quant-iT RiboGreen reagent was added and fluorescence signal was quantified using Tecan Infinite M200 Pro Multimode Plate Reader. Encapsulation efficiency was calculated as follows: $EE = \left[ {1 - \left( {\frac{{{\mathrm{unencapsulated}}\;{\mathrm{mRNA}}}}{{{\mathrm{total}}\;{\mathrm{mRNA}}}}} \right) \times 100} \right]$. LNPs and eLNPs containing siRNA were formulated using the same protocol as mRNA-containing nanoparticles. LNP and eLNP for smFISH and X-ray scattering experiments were prepared at the same molar ratios using NanoAssemblr Benchtop at a flow rate of 14 mL per min and lipid concentration of 12.5 mM. Nanoparticles were then dialyzed against PBS (pH 7.4) using Slide-A-Lyzer G2 dialysis cassettes (10k Da MWCO, Thermo Scientific) for 4 h at room temperature. The PBS was refreshed, and nanoparticles were left to dialyze overnight at 4 °C. Next, the particles were concentrated using Amicon Ultra centrifugal filters and characterized for hydrodynamic diameter (DynaPro, Wyatt Technology) and encapsulation efficiency.

### Cell culture

Full details of cell lines and their origins can be found in Supplementary Table 4. Cells were grown in appropriate growth media supplemented with 10–15% fetal bovine serum (Corning) and 5% Penicillin/Streptomycin (Corning). All cultures were grown in 37 °C incubators supplemented with 5% CO_2_ and were cultured according to suppliers’ instructions. Multiple primary and cancer cell lines were utilized to verify the robustness of our data and to prevent any false positives due to misidentified cell lines listed in The ICLAC Register of Misidentified Cell Lines.

### Synthesis and characterization of β-sitosterol conjugates

Different amino acid conjugates of β-sitosterol at the C-3 position were synthesized via carbodiimide coupling^68^ (Supplementary Fig. 3a). Fluorenylmethyloxycarbonyl (Fmoc)-protected amino acids were taken (1 mmol) and added to a solution containing β-sitosterol (1 mmol), N-hydroxy succinimide (NHS,1.35 mmol), N-(3-Dimethylaminopropyl)-N′-ethylcarbodiimide (EDC, 1.35 mmol) in dimethylformamide (DMF) and acetonitrile (4:1). The reaction was carried out for 8 h at 65 ^o^C. After the reaction; The protecting groups were cleaved with piperidine and trifluoroacetic acid as shown in the synthetic scheme. Meanwhile, β-sitosterol-acetate was also synthesized (Supplementary Fig. 3b). Briefly acetic anhydride (1.6 mmol) was added dropwise to a solution of cholesterol/β-sitosterol (1.2 mmol) with constant stirring in DMF with nitrogen purging. After 2 h of stirring, the acetate derivative of sterol was purified by repeated washing in water. All the synthesized conjugates were purified, dried and lyophilized and characterized using Fourier Transform-Infrared Spectroscopy ((Nicolet™ iS™ 5 FT-IR Spectrometer with iD7 ATR accessory) (Supplementary Fig. 3c).

### Cryo-TEM sample preparation and imaging

Five microliters of sample was applied onto a glow-discharged, 400 mesh Cu grid with thin carbon film supported by lacey carbon substrate (Ted Pella). Grids were blotted for 3 s at 22 °C and 100% humidity, then plunged into liquid ethane using a Vitribot Mark IV (FEI). Movie stacks were recorded with a K2 Summit camera (Gatan) in counting mode with a magnification of ×165,000 (0.53 Å per pix) on a Talos Arctica microscope (FEI) operated at 200 kV. Movie frames were gain normalized, aligned and summed using MotionCor2 in 5 × 5 patches with dose weighting^69^.

### X-ray scattering experiments

All the X-ray scattering experiments were performed using an in-house small-angle X-ray scattering instrument, SAXSpoint 2.0, from Anton Paar. X-rays of wavelength of 0.154 nm were generated from a Primux 100 micro X-ray source. The scattered intensity was measured using a two-dimensional (2D) EIGER R series CMOS detector from DECTRIS at a sample to detector distance of 575 mm. The 2D data was then circularly averaged, yielding the one-dimensional (1D) profile with *q* ranging from 0.06 nm^−1^ to 4 nm^−1^, where *q* $\left[ {q = \frac{{4\pi }}{\lambda }{\mathrm{sin}}\left( {\frac{\theta }{2}} \right) - } \right]$ is the wave vector, with *λ* and *θ* being the wavelength and scattering angle, respectively. The 1D data was further corrected for sample transmission and buffer background.

### Transfection

Cells were plated in white, clear-bottom 96-well plates. The number of cells plated per well varied by cell type: all diploid adherent cells (HeLa, human patient fibroblasts, mouse embryonic fibroblasts) were plated at 4000 cells per well, and macrophages were plated at 80,000 cells per well. Cells were allowed to adhere and grow for 24 h prior to LNP transfection except for siRNA transfection where cells were allowed to settle and adhere for 2 h prior to transfection. Nanoparticles were added dropwise on top of the media. Cell viability (CellTiter Fluor™ Cell Viability Assay, Promega), and luciferase expression (One-Glo™ Luciferase Assay, Promega) were measured after 24 h. Luciferase expression was normalized to cell viability.

### Twenty-four hours uptake study

In vitro HeLa transfection was performed using LNPs and eLNPs containing Cyanine-5 tagged EGFP mRNA (TriLink Biotechnologies). At 24 h post-transfection, media was aspirated, and cells were fixed in 4% paraformaldehyde in PBS for 15 min at room temperature. After fixation cells were washed three times with PBS and Hoechst-33258 (Life Technologies) was added (1:1000 in PBS) for nuclear staining. Cells were imaged at x20 magnification with a 455 x 455 µm field size, ten fields per well (Thermo Scientific Cellinsight NXT High-Content Screening Platform, Thermo Scientific HCS Studio: Cellomics Scan v.6.4.4 2013). Nuclei were defined as an object in channel 1 (386/23 nm) with a brightness threshold value of −0.366 and segmentation/shape threshold value of 10, allowing for identification of nuclei within tightly clustered cells and asymmetrical nuclei. The perinuclear space was defined as a region of interest ranging from 2 pixels into the nucleus to 18 pixels out from the edge of the nucleus. Cyanine-5 mRNA was imaged in channel 2 (650/14 nm) by filtering for objects with a total intensity between 0 and 9E7 (possible range: 0–10^12^) and an average intensity between 0 and 1.6^4^ (possible range: 0–6.5^5^). Uptake was calculated as mean perinuclear Cyanine-5 intensity divided by number of nuclei.

### Time-lapse live-cell LNP accumulation

Fluorescently labeled LNPs incorporating 0.1% Rhodamine/1,2-dioleoyl-sn-3-phosphatidylehanolamine (DOPE)^14^, and encapsulating Firefly Luciferase (FLuc) reporter mRNA were used to transfect HeLa cells (4k cells per well) in 96-well plates at 10 ng mRNA per well. HeLa cells were stained with DAPI (NucBlue™ Live, ThermoFisher) and CellTracker™ Deep Red (ThermoFisher) prior to LNP dosing, and live-cell imaging was done every hour for 24 h on Opera Phenix high-throughput spinning disk confocal (Perkin Elmer) equipped with environmental control using a 20x water immersion objective (NA 1.0). Image analysis was done in Harmony (PerkinElmer), individual cells were segmented using DAPI and CellTracker channels, and LNP accumulation in endocytic vesicles was quantified using spot segmentation in the Rhodamine channel. LNP accumulation at the single-cell level was quantified as cytosolic spots intensity sum normalized to cell area and reported as average per well (three wells per sample, five fields of view, and approximately 1000 cells per well).

### FACS analysis

293T-GFP-sgGFP cells were plated in 24-well plate at 60,000 cells per well and treated with LNP or eLNP delivering cas9 mRNA. Cells were then harvested 5 days later via trypsinization and placed in a single-cell suspension in PBS containing 10% FBS. Cell sorting was performed on a BD FACS-Aria Fusion equipped with a 488 nm laser and running BD FACS Diva v8.0.1 software. Forward scatter and side scatter gating was used to exclude debris. A single-cell gate based on forward scatter width and height was used to exclude aggregates. The GFP signal was detected through a 530/30 bandpass filter.

### High-resolution live-cell imaging

Three-dimensional trajectories of internalized LNPs and eLNPs were collected using 3D-Dynamic Photon Localization Tracking (3D-DyPLoTTo perform 3D-DyPLoT ^46,47^, a 640 nm excitation laser (Coherent OBIS, ~76 nW at the focus) is focused onto the sample by an objective lens (Zeiss Plan Apo, 100x NA=1.49) and is deflected in a 3D pattern by a 2D electro-optic deflector (ConOptics) and a tunable acoustic gradient (TAG) lens (TAG Optics)^70^. Fluorescence photons from the moving probe are collected by the objective lens and on focused through an emission filter onto a single-photon counting avalanche photodiode (Excelitas SPCM-ARQH-15). The time arrival of each photon is used to calculate the real-time 3D position of the moving probe within the laser scan using a field programmable gate array (NI-7852R). The real-time position is then used to drive a piezoelectric nanopositioner (MadCity Labs Nano-PDQ (XY) and Nano-OPHS (Z)) to hold the fluorescent target in the objective lens focal volume. This allows continuous 3D tracking with temporal resolution limited only be the photon emission rate. The readout of the nanopositioners is read out at 100 kHz. All trajectories shown are sampled every 1 millisecond. For LNP and eLNP tracking, nanoparticles were introduced to HeLa cells at 50% confluency in live-cell imaging buffer (Thermo Live-Cell Imaging Solution). The particles were allowed to internalize for 1 h, after which the cells were rinsed with PBS and returned to live-cell imaging buffer. The sample was then transferred to the microscope stage where it was maintained at 37 ^o^C for the duration of the tracking experimental with a coverslip heater (Bioscience Tools TC-CSC-18). Internalized particles are then tracked for 1–3 h from the start of the incubation.

To quantify linear transport, a process was adapted from Wagner et al.^49^, which quantifies the efficiency of transport. The equation used for quantification is shown below:

$$E_j = \frac{{\left( {x_{j + w} - x_j} \right)^2}}{{\left( {w - 1} \right){\mathrm{\Sigma }}_{i = j}^{j + w}\left( {x_i - x_{i - 1}} \right)^2}}$$

*E*_*j*_ is the linear efficiency at a point *j* within the trajectory, where the particle position is given by *x*_*j*_ a particle, which moves unidirectionally along a completely linear path would have a linear efficiency of 1. A particle that randomly diffuses would have a linear efficiency of 0. The linear efficiency was calculated over a sliding window (*w*) of 10 s for each point in the trajectory and these values are then averaged over the entire trajectory to define a single efficiency for each tracked particle.

### Endosomal escape efficiency characterization

Endosomal escape efficiency was measured using single-molecule imaging, as previously described^14^. Briefly, fluorescently labeled LNPs incorporating 0.1% Rhodamine/DOPE, and encapsulating FLuc mRNA were used to transfect HeLa cells in 96-well plates (Greiner BIO-ONE SensoPlate) at 2.5 ng mRNA per well in 100 µL cell culture media containing 10% fetal bovine serum. Cells were incubated with LNPs for 4 h, after that the samples were fixed in 4% paraformaldehyde (Ted Pella) and imaged on the Opera Phenix spinning disk confocal (Perkin Elmer) using a 63x water immersion objective (1.15 NA). Single-particle imaging on glass substrates was used to normalize cellular uptake and to derive the number of LNPs internalized at the single-cell level (Supplementary Fig. 6b). Stellaris single-molecule FISH (smFISH, Alexa647), which detects both cytosolic mRNA and mRNA trapped in endocytic organelles, was employed to detect intracellular FLuc mRNA. mRNA molecules that egressed the endocytic organelles into the cytosol were identified through object-based image analysis using the electroporated sample as benchmark for single mRNA intensity (gray signal). The selected single mRNA objects are pseudo-colored in green, overlaid over the smFISH signal (red). To quantitatively compare the endosomal escape efficiency for the two LNP formulations, we computed the ratio between the number of cytosolic mRNA and the number of internalized LNPs at the single-cell level.

### Statistical analysis

All experiments were performed in triplicate (*n* = 3) unless specified otherwise, and the significance was determined using multiple *t*-test (Graphpad Prism) for all comparisons except human peripheral blood macrophage ex vivo transfection and uptake study. Significance of human peripheral blood macrophage ex vivo transfection was evaluated using unpaired *t*-test (Graphpad Prism). Significance of Cyanine-5 mRNA uptake study was determined using nonlinear regression with ANOVA (JMP Pro 13, SAS). *p ≤ 0.05, **p ≤ 0.01, ***p ≤ 0.001.

### Reporting summary

Further information on research design is available in the Nature Research Reporting Summary linked to this article.

## Supplementary information

Supplementary Information

Description of Additional Supplementary Files

Supplementary Movie 1

Reporting Summary

## Data Availability

Data supporting the findings of this study are available within this paper, its Supplementary files, and Source Data file or available from the corresponding author upon reasonable request. The source data underlying Figs. 1f, 2b–f, 4a–e, 5a–c and Supplementary Figs 2a, b, 3a–d and 6b are provided as a Source Data file. Any other relevant data are available from the authors upon reasonable request.

## Source data

Source Data
