# Author Correction: Naturally-occurring cholesterol analogues in lipid nanoparticles induce polymorphic shape and enhance intracellular delivery of mRNA

**DOI:** 10.1038/s41467-020-17025-7

**Published:** 2020-07-06

**Authors:** Siddharth Patel, N. Ashwanikumar, Ema Robinson, Yan Xia, Cosmin Mihai, Joseph P. Griffith, Shangguo Hou, Adam A. Esposito, Tatiana Ketova, Kevin Welsher, John L. Joyal, Örn Almarsson, Gaurav Sahay

**Affiliations:** 1 Department of Pharmaceutical Sciences, College of Pharmacy, Oregon State University, Robertson Life Sciences Building, 2730 Southwest Moody Avenue, Portland, OR 97201 USA; 2 Moderna Therapeutics, 200 Technology Square, Cambridge, MA 02139 USA; 3 French Family Science Center, Department of Chemistry, 124 Science Drive, Duke University, Durham, NC 27708 USA; 4 Department of Biomedical Engineering, Oregon Health and Science University, Robertson Life Science Building, 2730 Southwest Moody Avenue, Portland, OR 97201 USA

**Keywords:** DNA and RNA, Targeted gene repair, Oligo delivery

Correction to: *Nature Communications* 10.1038/s41467-020-14527-2, published online 20 February 2020.

In the original version of this Article, the graphs presented in Figs. 4a, 5b, c and Supplementary Figs. 2 and 3a, d incorrectly designated ‘LNP’ as red and ‘eLNP’ as blue. ‘eLNP’ should be represented in red, and ‘LNP’ should be represented in blue. This has been corrected in both the PDF and HTML versions of the Article.

