# Supplementary material for: Naturally-occurring cholesterol analogues in lipid nanoparticles induce polymorphic shape and enhance intracellular delivery of mRNA
Source: Nat Commun. 2020 Feb 20;11:983. doi: 10.1038/s41467-020-14527-2 (PMC7033178; doi:10.1038/s41467-020-14527-2)
Supplement: Supplementary file 1 — Supplementary Information [file 41467_2020_14527_MOESM1_ESM.pdf]

Naturally-occurring cholesterol analogues in lipid nanoparticles induce polymorphic shape and enhance intracellular delivery of mRNA

Patel et al.

Supplementary Fig. 1

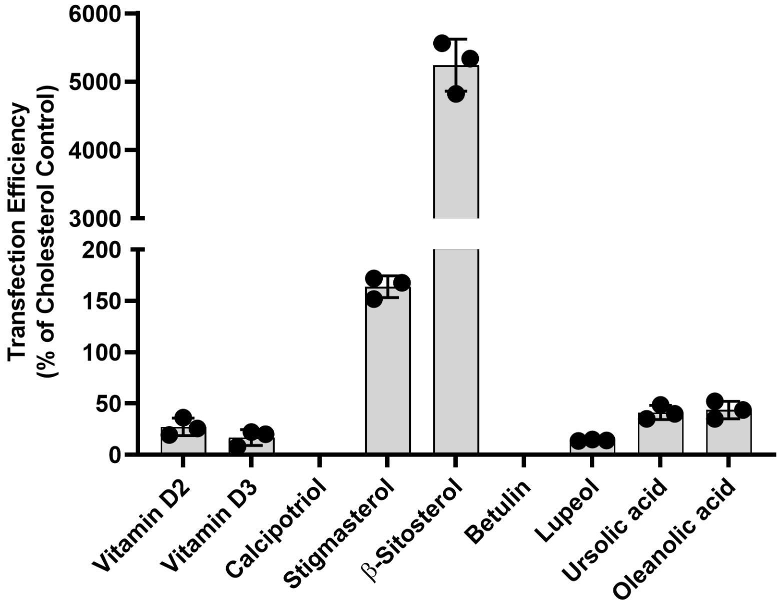

Supplementary Figure 1. Transfection efficiency of cholesterol analogues in initial screen.
Individual data points showing percentage transfection efficiency of cholesterol analogues used in the initial screen measured as luciferase expression relative to cholesterol control. (n = 3; mean ± SD). Source data are provided as a Source Data file.

Supplementary Fig. 2

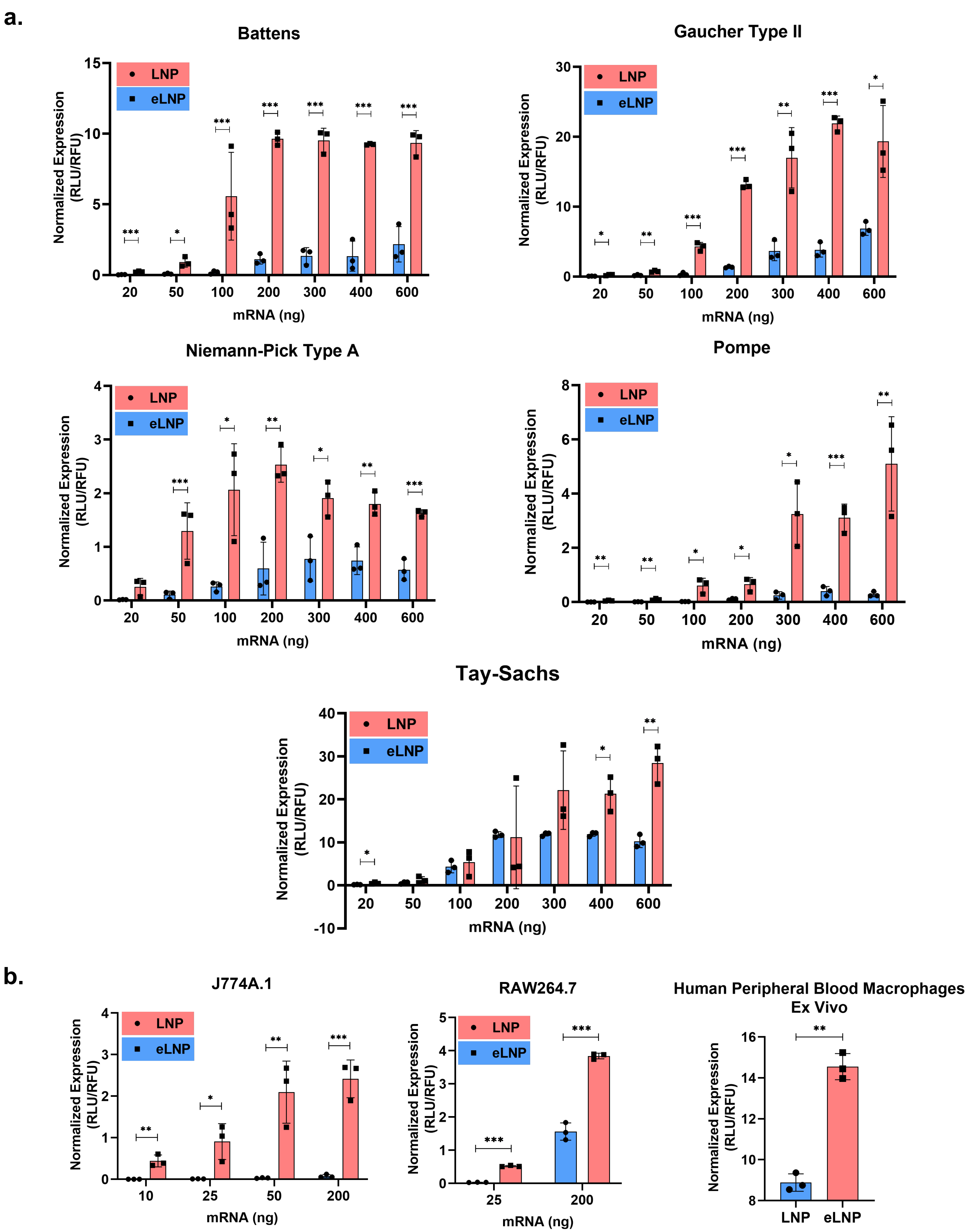

Supplementary Figure 2. Comparison of eLNP based gene delivery across a range of cell lines. Luciferase expression after transfection using LNP or eLNP in **(a)** 5 distinct human patient-derived fibroblast cell lines with lysosomal storage disorders, **(b)** three macrophage cell lines: RAW264.7 and J774A.1 cells, and peripheral blood macrophages from an apparently healthy human patient (1 µg mRNA per well). (n = 3; mean ± SD; *$p \leq 0.05$, **$p \leq 0.01$, ***$p \leq 0.001$; significance was determined using Multiple t-test. Significance of human peripheral blood macrophage ex vivo transfection was evaluated using unpaired t test.) Source data are provided as a Source Data file.

Supplementary Fig. 3

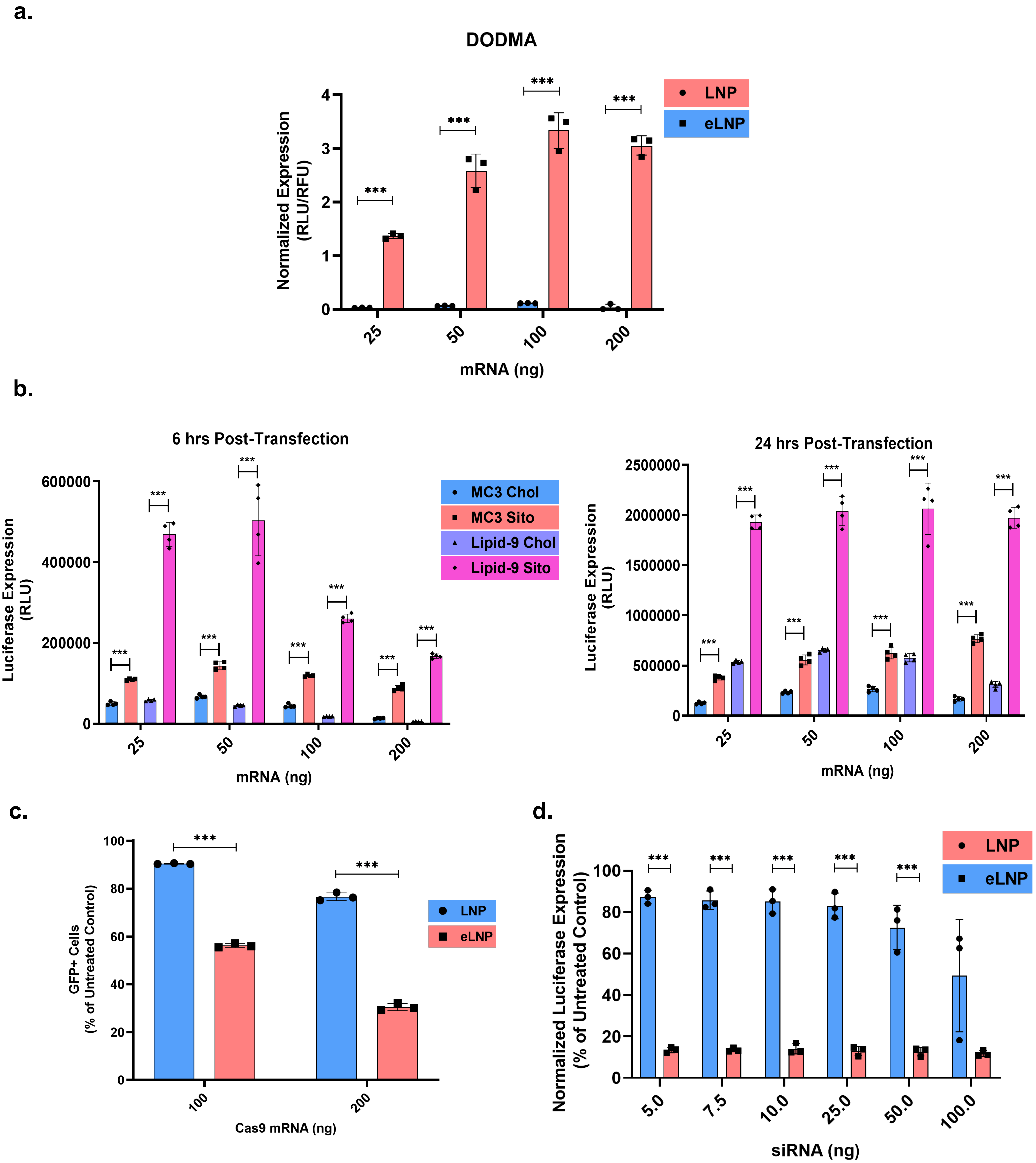

Supplementary Figure 3. β-sitosterol mediated transfection using different ionizable lipids or nucleic acids. **(a)** A different ionizable lipid (DODMA) was used to encapsulate mRNA in DODMA-eLNPs and DODMA-LNPs, and a dose-dependent (0-200 ng) transfection of HeLa cells was performed. **(b)** The luciferase expression of LNPs and eLNPs containing yet another ionizable lipid, Lipid 9, was also evaluated at 6- and 24-hr time point transfected at 25, 50, 100, 200 ng mRNA per well. **(c)** 293T-GFP-sgGFP were transfected with LNPs or eLNPs carrying Cas9 mRNA. FACS analysis was used to measure change in percentage of GFP-positive cells. **(d)** HeLa cells stably expressing luciferase protein were exposed to either LNP or eLNP packaged siRNA against luciferase. Expression (normalized to cell viability) is reported as percentage of untreated control ($n = 3$; mean ± SD; **p* ≤ 0.05, ***p* ≤ 0.01, ****p* ≤ 0.001; significance was determined using Multiple t-test.) Source data are provided as a Source Data file.

Supplementary Fig. 4

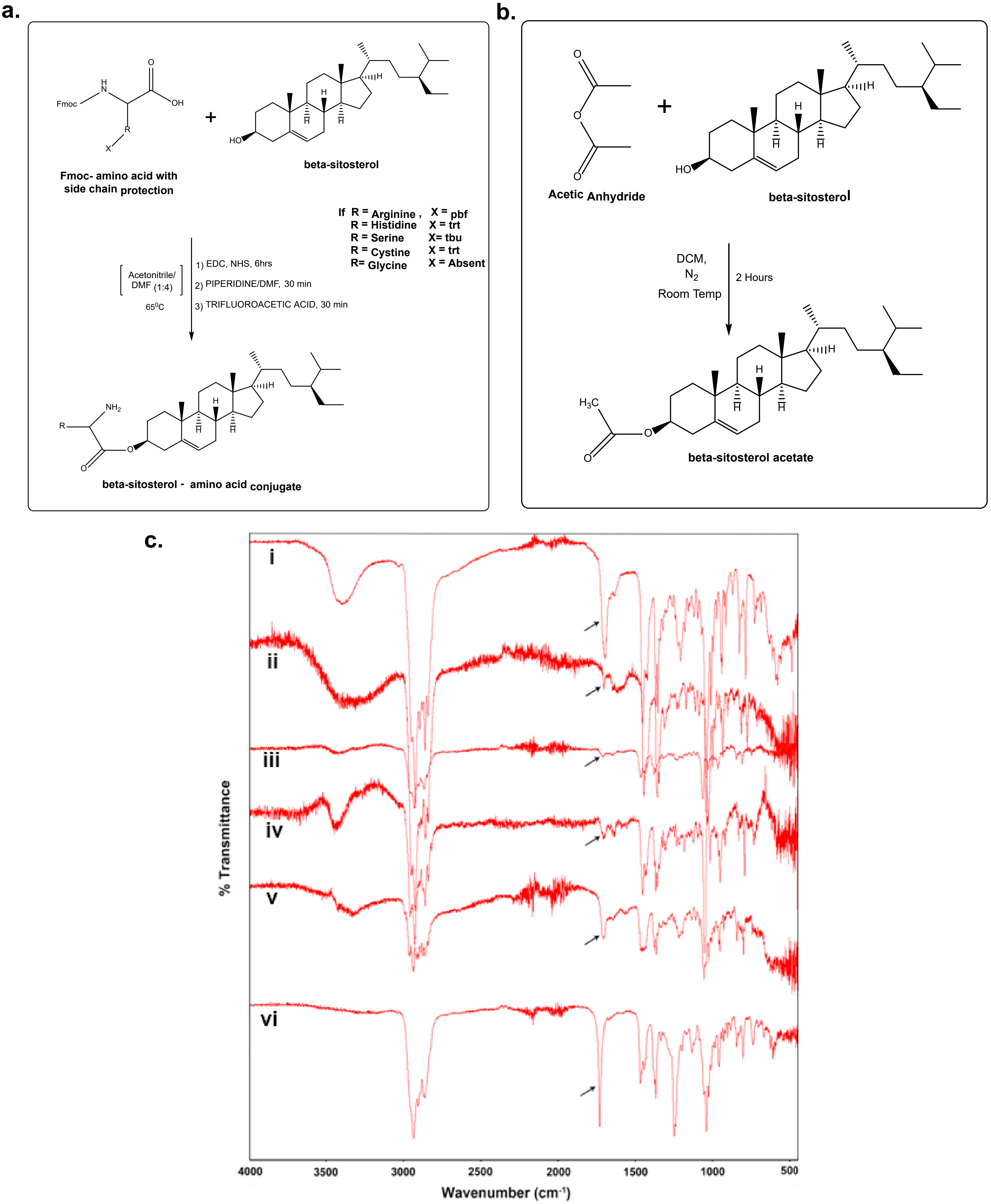

Supplementary Figure 4. Synthesis of C-3 modified sterols to investigate structure-function of eLNPs.
(a-b) Synthetic schemes for amino acid conjugates and acetyl conjugate of β-sitosterol respectively. **(c)** Corresponding FT- IR spectra of i) β-sitosterol-serine [1725 cm^{-1}], ii) β-sitosterol-glycine [1724 cm^{-1}], iii) β-sitosterol-histidine [1728 cm^{-1}] iv) β-sitosterol-arginine [1726 cm^{-1}] v) β-sitosterol-cysteine [1736 cm^{-1}] vi) cholesterol – acetate [1729 cm^{-1}], and vii) β-sitosterol-acetate [1734 cm^{-1}] . All analogues show ester peaks from 1724 cm^{-1} - 1736 cm^{-1} as shown by the black arrows. Polar group stretching (-OH and –NH2 groups) at 3050-3400 cm^{-1} in spectra i-v is absent for acetyl β-sitosterol by virtue of its structure.

Supplementary Fig. 5

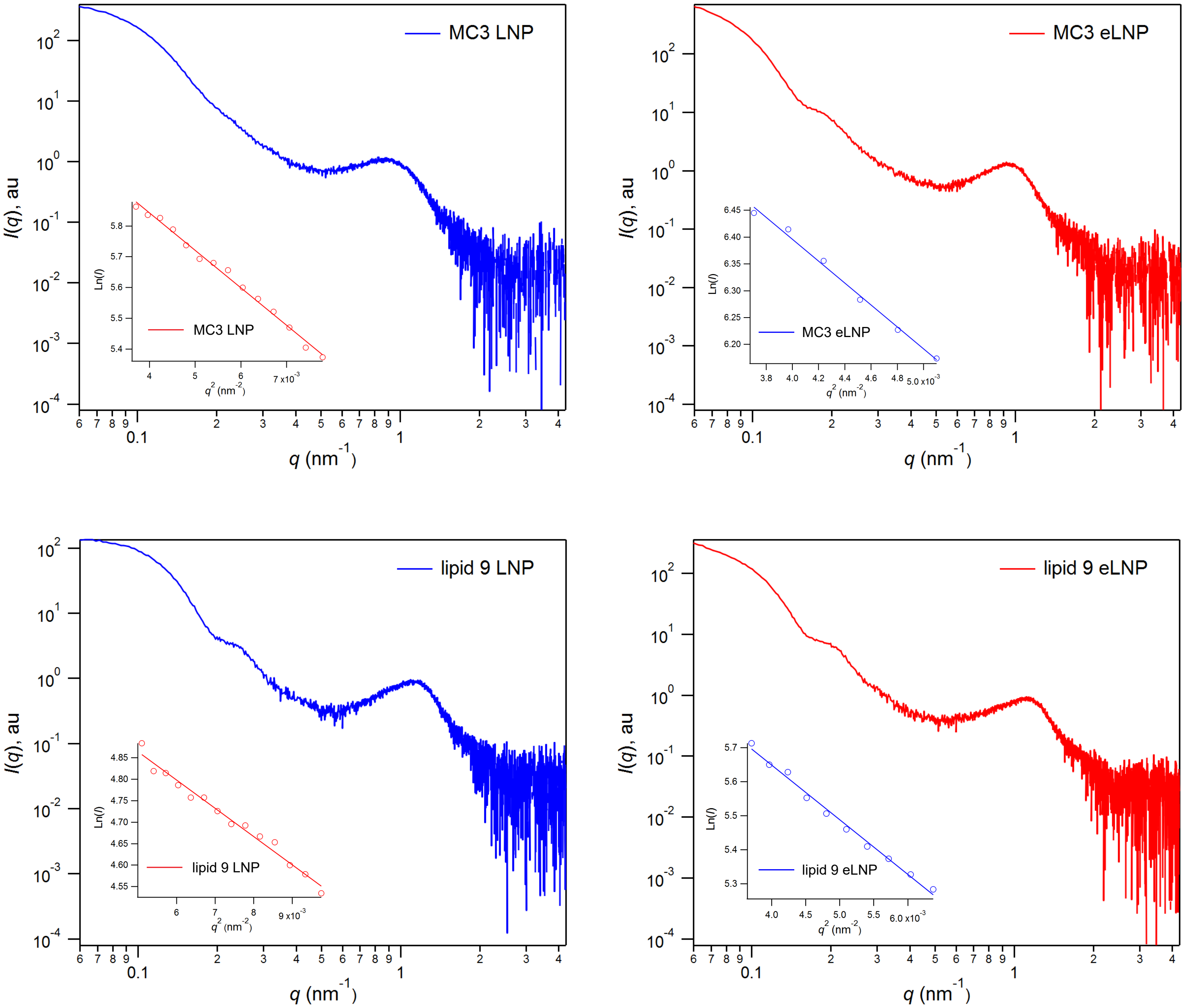

Supplementary Figure 5. Guinier approximation of MC3 or Lipid 9-based LNPs and eLNPs.
The Guinier approximation of each LNP is shown in the inserted figures, where Ln(*I*) is plotted against q^2. The slope of the Guinier plot is $Rg^{2/3}$. The resultant *Rg*s are 19.1 ± 0.23 nm, 24.2 ± 0.52, 14.0 ± 0.29, and 22.0 ± 0.36 nm for MC3 LNP, MC3 eLNP, lipid LNP, and lipid 9 eLNP, respectively. The linear fitting equations are $y = -121.79x + 6.33$ with R^2 of 0.9933 for MC3 LNPs, $y = -204.15x + 7.21$ with R^2 of 0.9929 for MC3 eLNPs, $y = -65.78x + 5.19$ with R^2 of 0.98, and $y = -160.99x + 6.29$ with R^2 of 0.9915.

Supplementary Fig. 6

a.

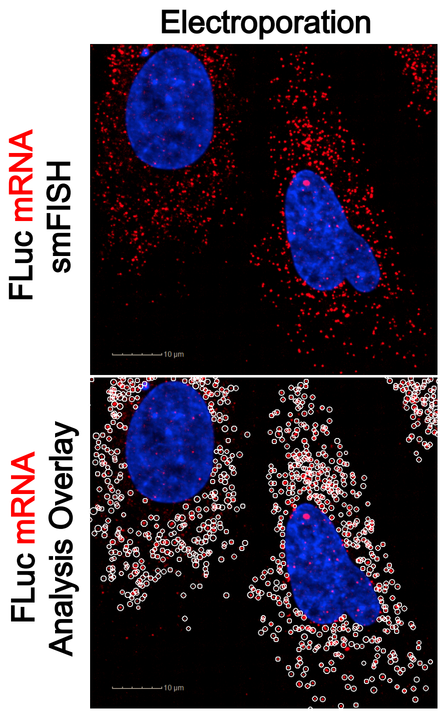

b.

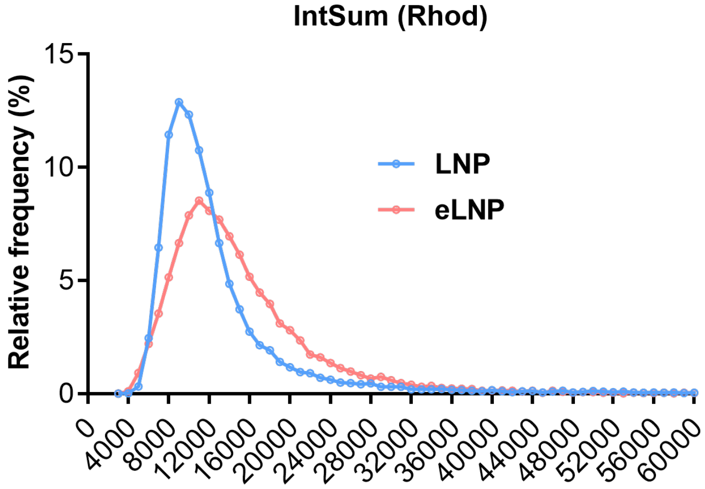

Supplementary Figure 6. Cytosolic localization of mRNA.
(a) Representative fluorescent images showing mRNA and image analysis after delivery with electroporation in HeLa cells. smFISH of free mRNA electroporated into HeLa cells (red signal) was used as a cytosolic mRNA control for quantitative image analysis of LNP internalization and cytosolic localization of mRNA (pseudo-colored in grey overlaid on the red smFISH signal). **(b)** Single LNP intensity distribution. Rhodamine labeled LNPs were diluted in DI water and incubated in glass substrate 96-well plates (Greiner BIO-ONE SensoPlate) for 5 min, after that the LNP containing solution was replaced with PBS and the adhered nanoparticles were imaged with Opera Phenix spinning disk confocal (Perkin Elmer) using a 63X water immersion objective (1.15 NA). For each sample multiple fields of view were imaged, and sum intensity in the Rhodamine was extracted at the single particle level (min 103 particles per sample analyzed). Histogram analysis was used to extract peak intensity for each formulation, and this value was used to normalize intracellular LNP accumulation for cell based experiments. Source data provided as Source Data file.

Supplementary Table 1. SAXS data collection parameters

Instrument	In-house Anton Paar SAXSpoint 2.0
Detector	2D EIGER R series Hybrid Photon Counting (HPC) detector (Dectris)
X-ray source	Primux 100 micro x-ray source (Cu)
Sample holder	Quartz capillary with 1 mm in diameter
Wavelength (Å)	1.5418
q range (nm^{-1})	0.06 to 4
Exposure time (minute)	120
Lipid concentration (mg mL^{-1})	20
Temperature (ºC)	25

Supplementary Table 2. The linear fit results for the Guinier plot of Ln [$I(q)$] against q^2

	Slope	Intercept	R^2	R_g*q_{max}	R_g (nm)*
MC3 LNP	-121.79 ± 2.89	6.33 ± 0.02	0.9933	1.69	19.1 ± 0.23
MC3 eLNP	-204.15 ± 8.61	7.21 ± 0.04	0.9929	1.72	24.2 ± 0.52
Lipid 9 LNP	-65.78 ± 2.71	5.19 ± 0.02	0.9800	1.38	14.0 ± 0.29
Lipid 9 eLNP	-160.99 ± 5.26	6.29 ± 0.03	0.9915	1.76	22.0 ± 0.36

* Note that the standard deviation for R_g was calculated based on the standard deviation of the linear fit results

Supplementary Table 3. MC3 or Lipid 9 LNP and eLNP characterization.

Ionizable Lipid	Sterol	Encapsulation Efficiency (%)	Hydrodynamic diameter (nm)	Polydispersity Index
DLin-MC3-DMA	Cholesterol	99	81	0.09
	Sitosterol	98	102	0.11
Lipid 9	Cholesterol	99	83	0.07
	Sitosterol	96	103	0.12

Encapsulation efficiency of MC3 and Lipid 9 LNPs and eLNPs and their hydrodynamic sizes and polydispersity index (PDI) as determined using DynaPro (Wyatt Technology).

Supplementary Table 4. Cell culture growth media and cell line source.

Cell Line	Medium	FBS	Source
HeLa (Kyoto subtype)	DMEM	10%	CVCL_1922 (Kind gift from Langer Lab)
HeLa-Luc	DMEM	10%	HeLa cells — wild type, stable cells that co-express Luciferase and Renilla (Kind gift from Langer Lab)
293T (stably expressing both EF1a promoter-GFP and U6 promoter-GFP targeting sgRNA (sgGFP))	DMEM	10%	293T cells (ATCC) infected with lentivirus to stably express EF1a-GFP (Addgene 26777) and U6-sgGFP (Kind gift from Langer Lab)
RAW264.7	DMEM	10%	ATCC (TIB-71) (Kind gift from Langer Lab)
J774A.1	DMEM	10%	ATCC (TIB-67) (Kind gift from Langer Lab)
Peripheral blood macrophage	RPMI 1640	10%	StemCell (Cat# 70042)
NPA Human patient fibroblasts	EMEM	15%	Coriell (GM00406)
NPC1 Human patient fibroblasts	EEMDUM	15%	Coriell (GM23162)
NPC2 Human patient fibroblasts	EEMDUM	15%	Coriell (GM18445)
Tay-Sachs human patient fibroblasts	EMEM	15%	Coriell (GM00077)
Gaucher Disease human patient fibroblasts	EMEM	15%	Coriell (GM02627)
Battens disease human patient fibroblasts	EMEM	10%	Coriell (GM16485)
Pompe disease human patient fibroblasts	DMEM	15%	Coriell (GM20089)
Immortalized mouse embryonic fibroblasts (WT $NPC1^{+/+}$ and $NPC1^{-/-}$)	DMEM	10%	Kind gift from Langer Lab
