# Supplementary material for: Naturally-occurring cholesterol analogues in lipid nanoparticles induce polymorphic shape and enhance intracellular delivery of mRNA
Source: Nat Commun. 2020 Feb 20;11:983. doi: 10.1038/s41467-020-14527-2 (PMC7033178; doi:10.1038/s41467-020-14527-2)
Supplement: Supplementary file 2 — Description of Additional Supplementary Files [file 41467_2020_14527_MOESM2_ESM.docx]

**Description of Additional Supplementary Files**

**Supplementary Video 1:** Video showing representative 3D trajectories of LNP or eLNP inside a cell acquired using 3D-DyPLoT at a temporal resolution of 1 msec.
